# Role of dislocations in nitride laser diodes with different indium content

**DOI:** 10.1038/s41598-020-79528-z

**Published:** 2021-01-08

**Authors:** Agata Bojarska-Cieślińska, Łucja Marona, Julita Smalc-Koziorowska, Szymon Grzanka, Jan Weyher, Dario Schiavon, Piotr Perlin

**Affiliations:** 1 Institute of High Pressure Physics, “Unipress”, Sokolowska 29/37, 01-142 Warsaw, Poland; 2 TOP-GAN Limited, Solec 24/90, 00-403 Warsaw, Poland

**Keywords:** Lasers, LEDs and light sources, Optical materials and structures

## Abstract

In this work we investigate the role of threading dislocations in nitride light emitters with different indium composition. We compare the properties of laser diodes grown on the low defect density GaN substrate with their counterparts grown on sapphire substrate in the same epitaxial process. All structures were produced by metalorganic vapour phase epitaxy and emit light in the range 383–477 nm. We observe that intensity of electroluminescence is strong in the whole spectral region for devices grown on GaN, but decreases rapidly for the devices on sapphire and emitting at wavelength shorter than 420 nm. We interpret this behaviour in terms of increasing importance of dislocation related nonradiative recombination for low indium content structures. Our studies show that edge dislocations are the main source of nonradiative recombination. We observe that long wavelength emitting structures are characterized by higher average light intensity in cathodoluminescence and better thermal stability. These findings indicate that diffusion path of carriers in these samples is shorter, limiting the amount of carriers reaching nonradiative recombination centers. According to TEM images only mixed dislocations open into the V-pits, usually above the multi quantum wells thus not influencing directly the emission.

## Introduction

Gallium nitride based laser diodes with InGaN multi quantum wells serving as an active layer can emit light in the broad range of visible and UV spectrum, from UV-A to green. These devices were critical to developement of new optoelectronic systems like: laser car headlights, laser based light engines for projectors, various tools for printing and 3D printing as well as contributed to the improvement of existing technologies like super-DVD standard: BluRay. Problem of the influence of threading dislocations on the properties of nitride light emitters including laser diodes is one of the questions which have been discussed ever since the early days of nitride technology. The availability of native GaN substrates was scarce at the beginning of nitride history and consequently the majority of nitride optoelectronic devices were grown on sapphire. As the lattice mismatch between sapphire and GaN is substantial, these structures are characterized by high density of threading dislocations, typically between 5 × 10^8^ cm^−2^ and 1 × 10^9^ cm^−2^. In nitrides we distinguish three types of dislocations that differ with the Burgers vector *b*: edge-type (b = a), screw-type (b = c) and mixed type (b = a + c)^1^. Multiple studies report that some or all of them are playing a role of nonradiative recombination centers in GaN based devices^1–7^.

However, in spite of high dislocation density, light emitting diodes (LEDs) with InGaN quantum wells serving as an active region, turned out to be almost insensitive to the low crystallographic quality of these structures^4,8^. In fact, as for today, the visible nitride LEDs with external quantum efficiency exceeding 80%, were demonstrated^9^. The astonishingly low impact of dislocations on these devices was and still is broadly discussed. There are two explanations proposed, one initiated by Chichibu, assuming carrier localization due to In fluctuations (or short diffusion length) as a factor limiting the contact of injected carriers with dislocations^8^. The second way of reasoning was exemplified by the papers of Hangleiter et al. and Speck et al.^5,10^. It assumes the presence of an energetic barrier around dislocations existing due to V-pits formation in the active region. Thinning of the quantum wells results in higher local potential which prohibits the carriers from nonradiative recombination^10^.

However, InGaN laser diodes seem to be much more sensitive to dislocations than LEDs, forcing engineers to adopt a different approach. It was actually a paradigm of nitride laser technology that the reduction of threading dislocations density in InGaN laser diode structures is necessary for device perfectness and longevity. This claim was surprisingly more matter of a “common knowledge” than a well-documented and experimentally based model. In fact, the data on the influence of the dislocation density on the lifetime of InGaN laser diodes was gathered long time ago and never truly reconfirmed^11–13^. The present trend to develop the laser diode on various platforms like Si or even metals makes the reconciliation of the role of dislocation important and timely!^14,15^.

The direct inspiration to this work was the observation made during the routine characterization of the laser diodes structures (epiwafers) grown by MOVPE method on GaN bulk substrates and their counterparts grown on sapphire. It turned out that the structures emitting in the blue region (440–450 nm) were very efficient (strong light emission) independently from the substrate material. In contrast, the UV-A devices (wavelength shorter than 400 nm) emitted substantial amount of light only when grown on low defect density GaN. This paper is dedicated to give physical meaning of this observation. We apply a number of analytical tools to study this effect, including cathodoluminescence and multiple microscopic techniques like SEM, TEM and AFM.

## Investigated structures

In the first part of the experiment we compare a set of laser diode structures (unprocessed epiwafers) emitting in the spectral region from 385 to 455 nm. All samples were fabricated as GRINSCH (Graded index separate confinement heterostructure) devices^16^. Structures were grown on two types of substrates—bulk GaN and sapphire. The most important difference between those two substrates is dislocation density, equal to 10^5^–10^6^ cm^−2^ for GaN and 10^8^–10^9^ cm^−2^ for sapphire. Fully processed, laser diodes grown on gallium nitride substrate (we did not processes structures on sapphire to laser diodes) were characterized by the threshold current density of around 2 kA*cm^−2^ and differential efficiency of 1.1 W/A, independently from their wavelength (for ridge dimensions of 1000 μm × 3 μm).

We observed that well optimized laser diodes, operating between 390 and 460 nm, maintain their threshold parameters.

In the second part of the study, to avoid effects caused by the p-type layers growth, we studied structures consisting of the active region capped with just thin GaN layer. These samples were grown on sapphire substrates and emitted light in the wavelength range 395–466 nm.

**Figure 1:**
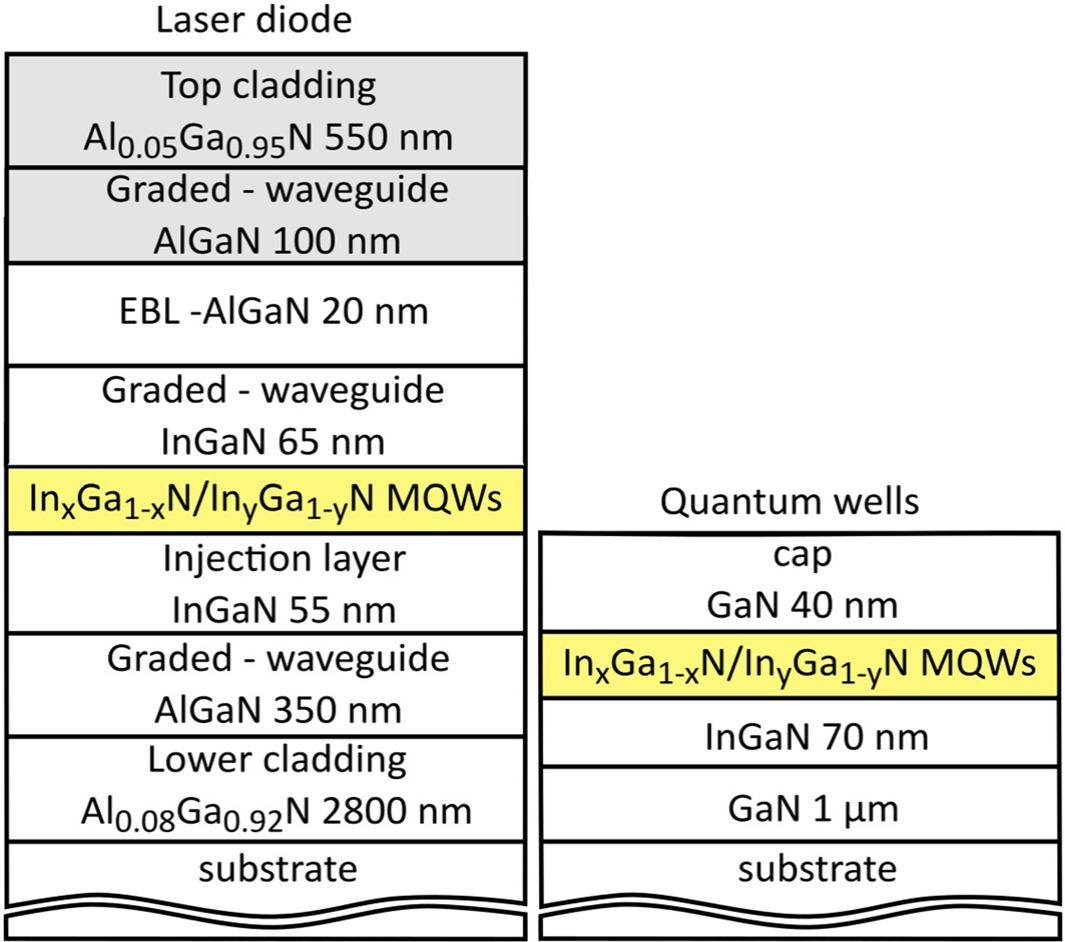
Simplified schemes of investigated epitaxial structures.

All investigated structures were grown using metal organic vapor phase epitaxy (MOVPE). Simplified general schemes of studied structures are shown in Fig. 1.

## Results

### Electroluminescence

**Figure 2:**
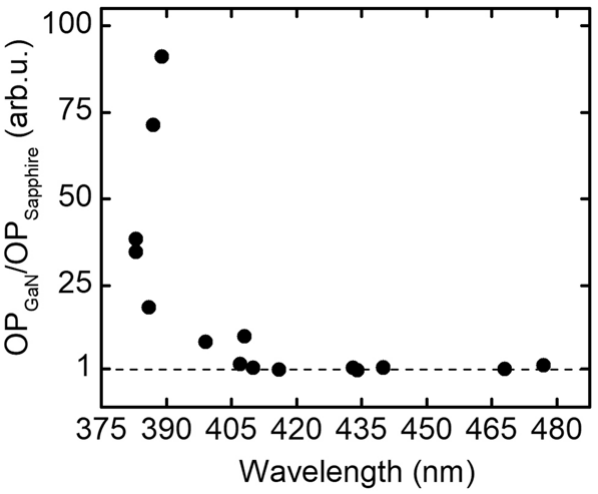
Ratio of electroluminescence signal obtained for laser diode structures grown on bulk GaN and sapphire, emitting in the range from λ = 383 nm to λ = 477 nm.

One of the standard characterization techniques of the unprocessed nitride laser diode epitaxial structures is the measurement of intensity of electroluminescence. In order to choose best growth procedures and for quality control, we always grow laser diode structures on two different types of substrates, bulk GaN and sapphire. We observed that samples with high in composition of the quantum wells are characterized by similar, high electroluminescence intensity, regardless of the substrate type. For short-wavelength emitting structures we observe significant difference in electroluminescence signal. In this case, intensity of light is always much lower for samples grown on sapphire. This very striking result is shown in Fig. 2.

Figure 2 shows that in the spectral region between roughly 410 and 480 nm, the ratio of the emitted light from the structure on sapphire and on bulk GaN is close to 1. For the shorter wavelength we observe a kind of threshold below which the emission of the structures grown on sapphire substrates rapidly decreases. At around 390 nm the emission from sapphire based material is almost hundred times weaker than from the one grown on bulk GaN. One should remember that the ratio shown in Fig. 2 is very scattered once the intensity of the light emission from the structures grown on sapphire becomes close to zero.

Looking for an explanation of observed difference in light intensity on wavelength dependence in case of laser diode structures grown on different substrates, we performed detailed analysis of subthreshold L-I curves of multiple devices.

**Figure 3:**
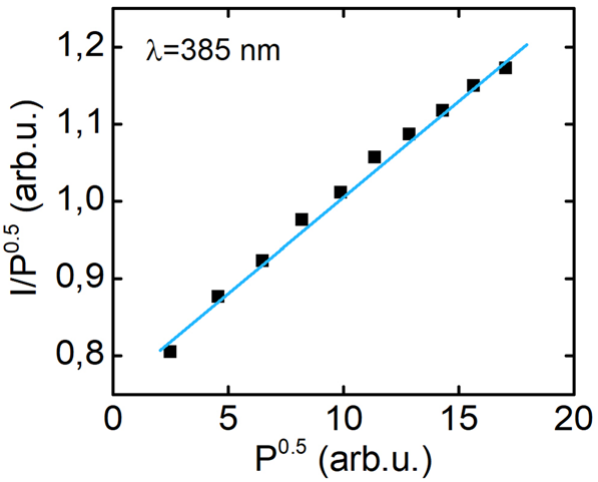
Exemplary light – current characteristic in $\sqrt{P}$, $\frac{I}{\sqrt{P}}$ coordinates.

In this experiment, current was varied from 1 to 20 mA and light was collected in direction perpendicular to the samples’ surface. We assume that for such small currents we can neglect Auger recombination. Therefore, we take into account just two mechanisms of carrier recombination: Shockley–Read–Hall-like nonradiative recombination and bimolecular radiative recombination. Then the participation of different recombination mechanisms in total recombination in reference to carriers density (N) and current density (j) can be described by the following equation^17^:

1 $$\frac{{j \eta_{j} }}{e} = AN + BN^{2}$$

where A and B are nonradiative and radiative recombination rates and ${\eta }_{j}$ is the injection efficiency. As optical power (P) is proportional to BN^2^, we can transform Eq. (1) into:

2 $$\frac{I}{\sqrt P } = a + b\sqrt P$$

where $a=\frac{qVA}{{\eta }_{j}\sqrt{kB}}$ and $b=\frac{qV}{{\eta }_{j}k}$ , with *k* being the light coupling constant to the detection system in the experiment, V is the volume of the active area and *q* is the charge of an electron. Plotting light–current characteristics in $\sqrt{P}$, $\frac{I}{\sqrt{P}}$ coordinates allows us to observe a linear dependence, as shown in Fig. 3. The experimentally observed linearity of this dependence confirms that we can neglect the cubic term corresponding to Auger nonradiative recombination in Eq. (1).

**Figure 4:**
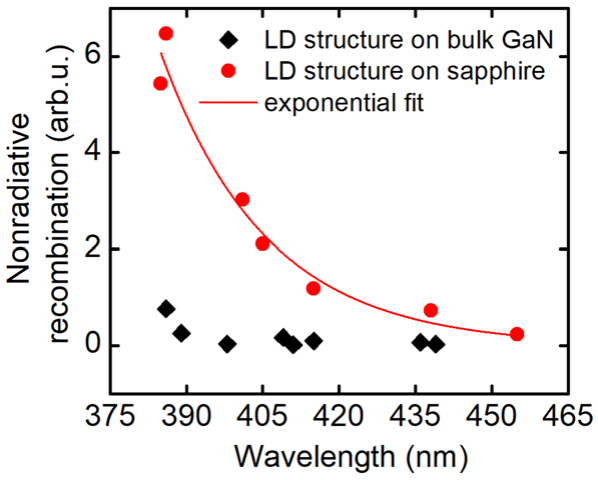
Nonradiative recombination as a function of emission wavelength for laser diode structures grown on bulk GaN and sapphire.

Using appropriate fitting, we determined parameter *a*, proportional to $\frac{A}{\sqrt B }$ thus giving us qualitative information about nonradiative recombination level. As there is no evolution in the emission from various structures grown on bulk GaN, we presume that in case of the structures grown on sapphire, they should differ only in A parameter and B should stay relatively constant. Figure 4 presents parameter *a* versus emission wavelength, for structures grown on two types of substrates. We see explicit difference between those two plots. In case of laser diodes grown on bulk gallium nitride parameter *a* is low and constant in almost whole wavelength range, with just small increase at the shortest wavelength. For laser diodes grown on sapphire, we observe an exponential growth of *a* parameter with the decreasing wavelength (decreasing In content).

### Cathodoluminescence

To explain the origin of observed phenomena, we investigated two representative structures grown on sapphire, emitting at 382 nm (sample “LD1”) and 438 nm (sample “LD2”). Images obtained for both samples using Atomic Force Microscope (AFM) were identical, showing atomic steps and same amount of dislocations. As these samples have thick p-type layers we decided to look closer at the active region.

For that purpose we grew structures with just one thin InGaN layer after the quantum wells, eliminating the effects of p-type layers epitaxial growth. These samples emitted light at 395 nm (“QW1”) and 466 nm (“QW2”). Their simplified scheme is shown in Fig. 1. New structures were characterized using cathodoluminescence and transmission electron microscope TEM.

**Figure 5:**
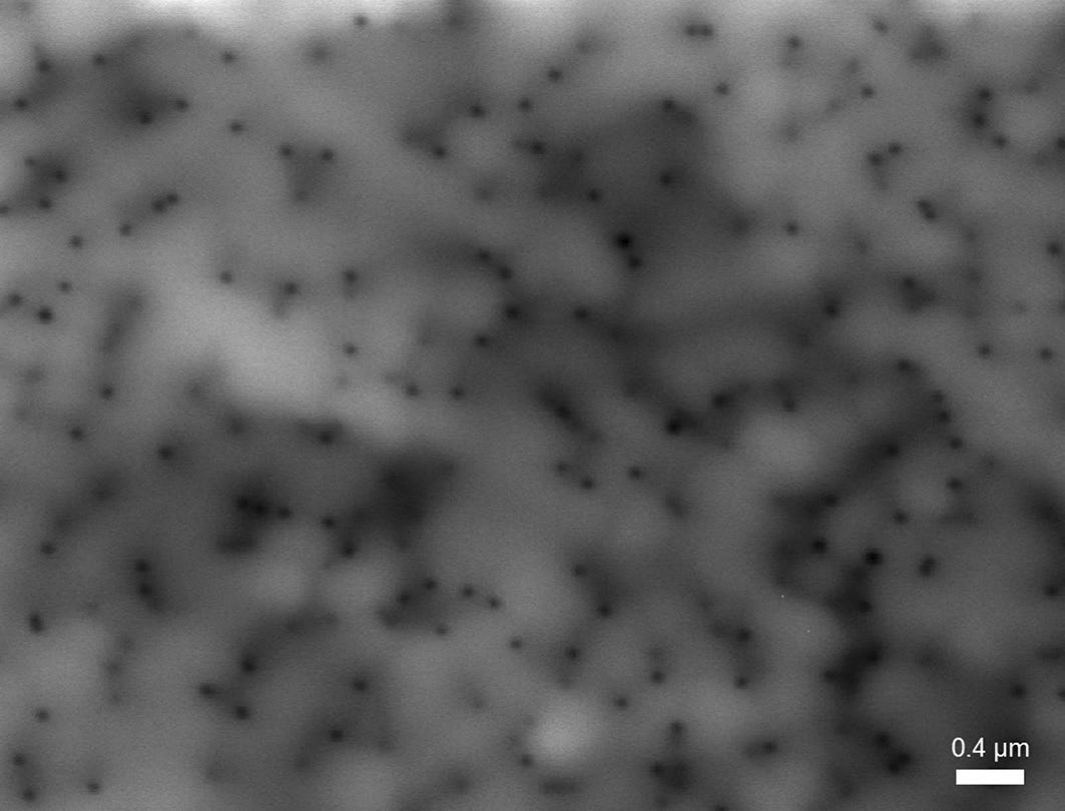
Cathodoluminescence map of sample emitting at λ = 395 nm.

**Figure 6:**
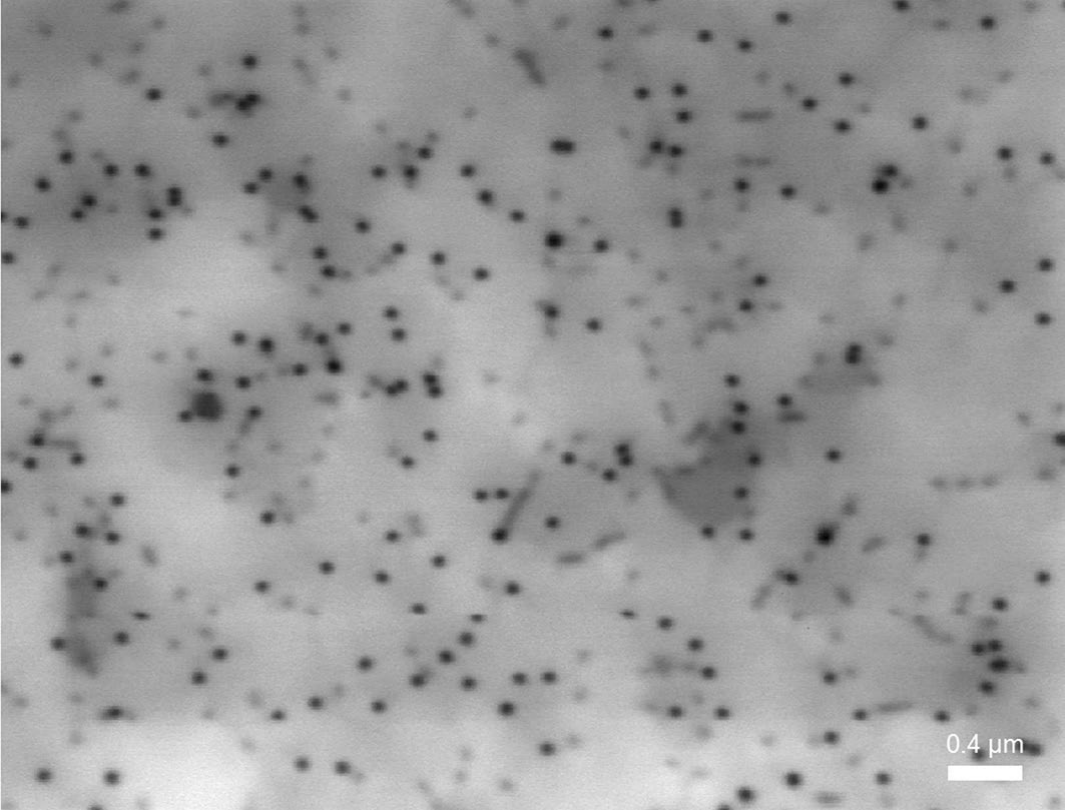
Cathodoluminescence map of sample emitting at λ = 466 nm.

**Figure 7:**
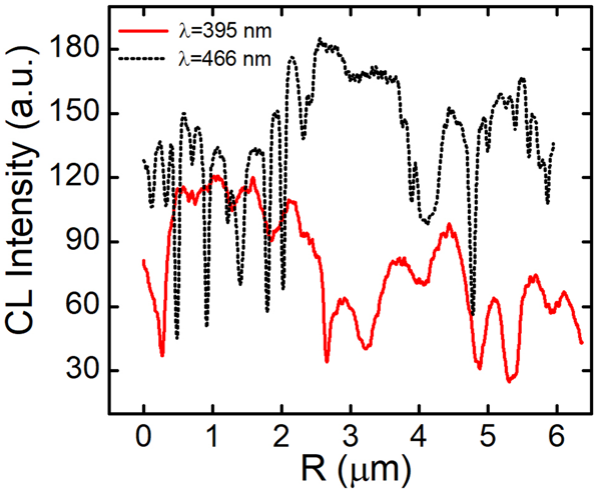
Cathodoluminescence intensity as a function of position for QWs emitting at λ = 395 nm and λ = 466 nm.

**Figure 8:**
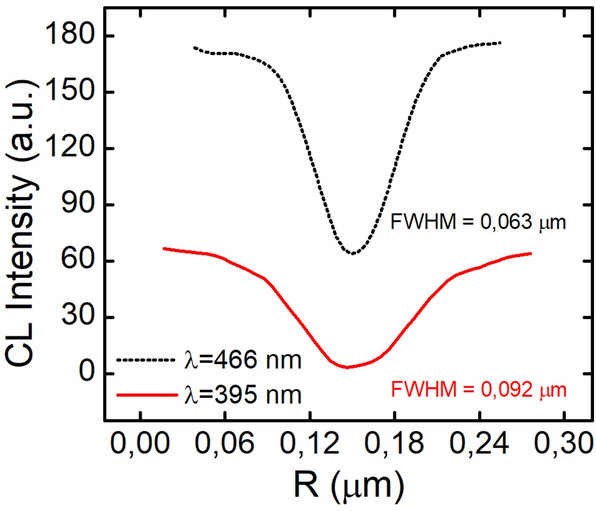
Cathodoluminescence intensity for one dislocation for QWs emitting at λ = 395 nm and λ = 466 nm.

Cathodoluminescence is one of the most useful and popular techniques for identifying dislocations as nonradiative recombination sources^1,18–22^. Figures 5, 6 show cathodoluminescence maps obtained for samples QW1 (Fig. 5) and QW2 (Fig. 6). There is a striking difference in brightness of taken images and less obvious in the dark points size. To make it clearer, in the Figs. 7, 8 we plotted the integrated line profiles of the CL maps and of chosen single dark points. Both of them show that laser diode structure emitting at longer wavelength is characterized by smaller dark points (FWHM = 0.063 nm in comparison to FWHM = 0.092 nm for the short-wavelength sample). There is also some difference in determined dark points densities, which are $d_{dp\_QW1} = 6.6 \times 10^{8}$ cm^−2^ and $d_{dp\_QW2} = 1.25 \times 10^{9}$ cm^−2^. However, number of dislocations in the epitaxial structure depends mostly on their density in the GaN/sapphire templates, being thus independent of quantum wells growth step.

Figure 7 indicates, that in the short wavelength structures, the average level of light emission is lower, especially between the dislocations (grey regions in Fig. 5). That can be explained by longer diffusion length of carriers. However, the size of dislocation spots itself is comparable for all structures (up to 50% difference) and therefore cannot be directly connected with the diffusion length of carriers. The size of dark points is most likely related to the shape of potential barriers around dislocation.

Obtained data confirms our previous observations of electroluminescence intensity on wavelength dependence and their interpretation attributing light decrease to increase in nonradiative recombination due to dislocations.

### Temperature dependence of electroluminescence intensity

**Figure 9:**
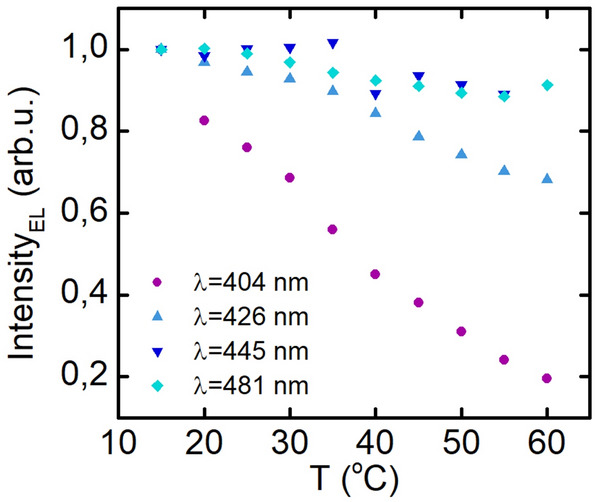
Intensity of electroluminescence on temperature dependence for laser diode structures emitting at different wavelengths.

In the next step we investigated the temperature dependence of intensity of electroluminescence for laser diode structures grown on sapphire with different indium composition of the active region, emitting in the range from λ = 404 nm to λ = 481 nm. The temperature was varied from 15 to 60 °C with 5 °C step, and the operation current was equal to I = 100 mA. Obtained results are shown in the Fig. 9. We observe that structures emitting at longer wavelength are characterized by better thermal stability than the short-wavelength devices. This result indicates more effective carriers localization in structures with higher In content. That may be caused by more pronounced indium fluctuations^8^ and less effective thermal escape of electrons from the deeper quantum wells^23^. These two factors limit both the diffusion length and number of carriers that can recombine nonradiatively.

These results are in compliance with our previous research performed on LDs grown on bulk GaN^23^.

### Dislocation identification

To identify type of dislocations that contribute most to nonradiative recombination we investigated chemically etched laser diode structures (LD1, LD2) and both QW structures (QW1, QW2).

**Figure 10:**
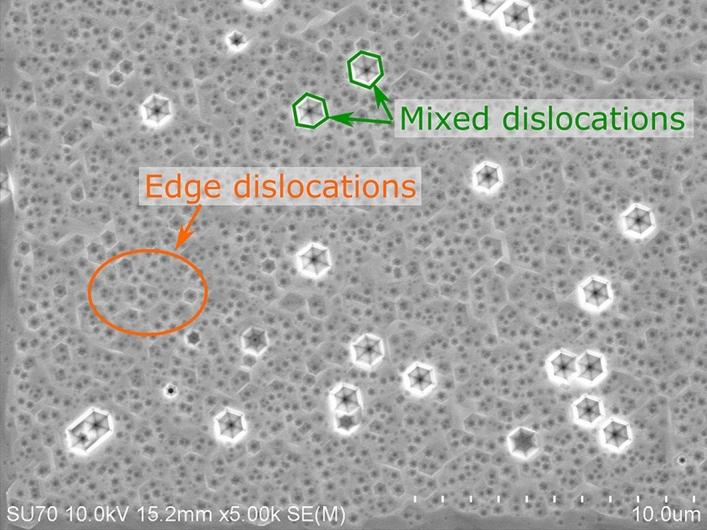
SEM picture of etched laser diode structure.

We performed chemical etching of the LD1 and LD2 to reach the level of the InGaN waveguide^24,25^. Samples were then studied using Scanning Electron Microscope (SEM). As results obtained for both structures had no significant differences, we show only one SEM picture (Fig. 10). Due to selective etching all the threading dislocations are visible, allowing us to identify their type and estimate dislocation density. We assign the big, hexagonal pits to mixed dislocations (Burger’s vector a + c) and the small ones to edge dislocations (a), which is consistent with TEM results, described in the next paragraph. Screw dislocations in our samples are extremely rare and therefore their input to the nonradiative recombination can be neglected. This is consistent with the results shown by Albrecht et al.^22^. Density of each type of dislocations are: $d_{a + c} = 5.16 \times 10^{6}$ cm^−2^ and $d_{a} = 4.76 \times 10^{8}$ cm^−2^. That gives total dislocations density equal to $d_{tot} = 4.81 \times 10^{8}$ cm^−2^. Comparing these values to the dark points density in cathodoluminescence images (Figs. 5 and 6) we notice that they are the same order of magnitude. This indicates that nonradiative recombination is related to either to all types of dislocations or just the edge ones (which constitute the overwhelming majority of total amount of dislocations).

**Figure 11:**
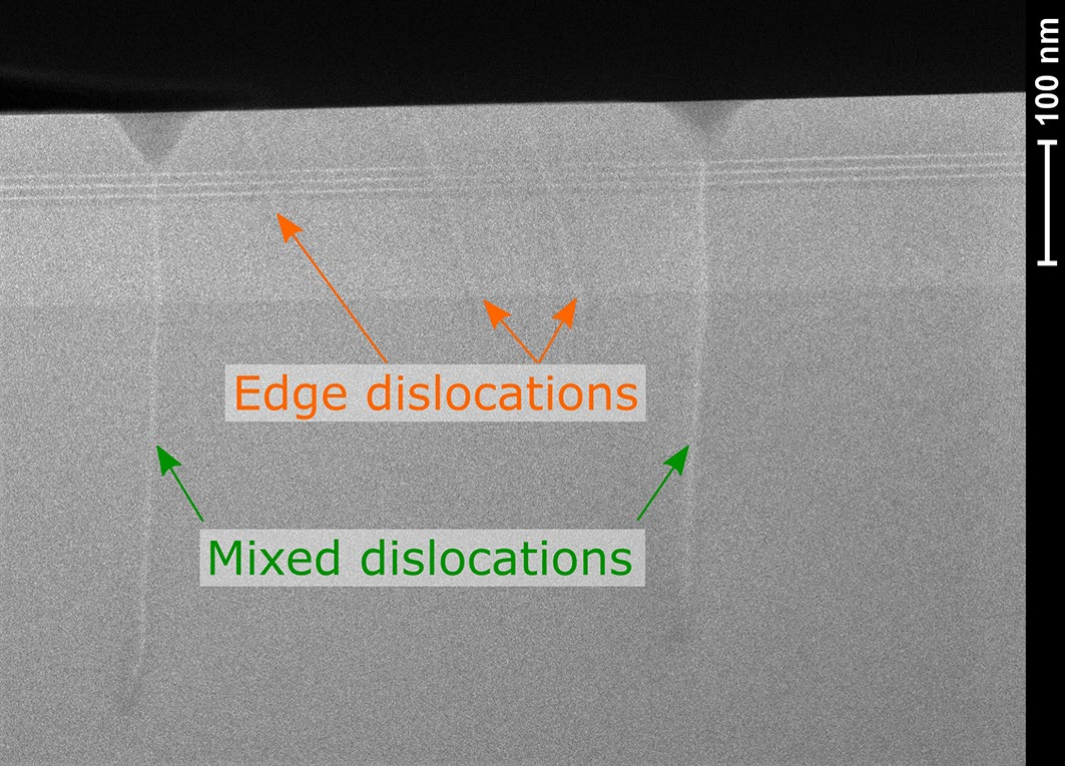
TEM picture of QW structure with visible edge and mixed dislocations.

To see if nonradiative recombination takes place due to opening dislocations into the V-pits in the quantum wells, resulting in their decomposition, QW1 and QW2 structures were investigated using TEM microscope. As results once again were similar for both samples, we present just one of them, in the Fig. 11. It shows that mixed dislocations open into the V-pits after the last quantum well, while edge dislocations do not. None of them seem to cause quantum wells decomposition, what justifies similar dark spots diameter in cathodoluminescence images (Figs. 5 and 6). At the same time we do not exclude possible QW degradation during the p-type growth, linked to diffusion along certain types of dislocations, especially in structures with high In content. The presence of V defects results in formation of largest pits on mixed dislocations during defect selective etching (Fig. 10).

TEM results indicate that we do not observe the dislocation screening effect proposed by Hangleiter et al.^10^. In this model potential barrier is formed around the dislocation due to V-pit formation in the active region, which causes thinning of the QWs. In our case, independently of the layer In composition, V-pits are formed above the active region and only for minor part of dislocations. Therefore, we assign differences in light emission from samples with varying In composition grown on sapphire to differences in diffusion path of carriers.

## Summary

In this work we investigated the influence of dislocations on nonratiadive recombination in nitride laser diode structures with different indium content.

Analysis of electroluminescence showed that nonradiative recombination decreases exponentially with the increase in the indium content in case of LDs grown on sapphire substrate (with high dislocation density). Structures grown on bulk GaN are characterized by low level of nonradiative recombination independent of In content.

Further studies of samples grown on sapphire were aimed at identification of nonradiative recombination sources. Density of dark points in CL images suggest that mainly edge dislocations are sources of nonradiative recombination in the whole investigated wavelength range. At the same time, we observed that in structures with higher indium content, nonradiative recombination of carriers takes place in the smaller area around the dislocation. These results, together with differences in temperature dependence of electroluminescence of samples emitting at different wavelength, suggest that diffusion length of carriers depends on the In content of the active layers.

Characterization of samples with TEM microscope revealed that in our structures, independently on the In composition, mixed dislocations open into V-pits above the last quantum well. Consequently, we do not observe effect of potential barrier around dislocation caused by local thinning of the QWs as assumed in Hangleiter model^10^. Therefore, increase in nonradiative recombination for UV laser diode structures may be due to less effective carriers localization (longer diffusion length).

## Methods

Density and type of dislocations (edge/mixed/screw) were established by defect selective etching (DSE method: molten KOH − NaOH eutectic + 10% of MgO^24,25^). After etching samples were examined in differential interference contrast (DIC) optical microscope (Nikon Eclipse 80i) and in scanning electron microscopy (SEM).

Cathodoluminescence and SEM pictures were measured using Hitachi SU-70 scanning electron microscope equipped with Horiba Jobin Yvon optical detection system.

TEM pictures were taken with Transmission Electron Microscope FEI Tecnai G2 F20 S-TWIN.
